# Metabolic Imaging in B-Cell Lymphomas during CAR-T Cell Therapy

**DOI:** 10.3390/cancers14194700

**Published:** 2022-09-27

**Authors:** Flavia Linguanti, Elisabetta Maria Abenavoli, Valentina Berti, Egesta Lopci

**Affiliations:** 1 Nuclear Medicine Unit, Department of Experimental and Clinical Biomedical Sciences “Mario Serio”, University of Florence, 50134 Florence, Italy; 2 Nuclear Medicine Unit, IRCCS—Humanitas Research Hospital, Via Manzoni 56, 20089 Rozzano, Italy

**Keywords:** B-cell lymphoma, non-Hodgkin lymphoma, CAR-T, FDG, PET/CT, response, adverse events

## Abstract

**Simple Summary:**

Chimeric antigen receptor–engineered T cells are an innovative therapy in hematologic malignancies, especially in patients with refractory/relapsed B-cell lymphomas. Few studies have analyzed the role of [^18^F]FDG PET/CT in this field; this review aims to illustrate the literature data and the major findings related to [^18^F]FDG PET/CT use during CAR-T cell therapy in B-cell lymphomas, focusing on the prognostic value of metabolic parameters, as well as on response assessment. Furthermore, this work shows in detail the specific adverse events during CAR-T cell therapy and the role of [^18^F]FDG PET/CT imaging in their occurrence.

**Abstract:**

Chimeric antigen receptor–engineered (CAR) T cells are emerging powerful therapies for patients with refractory/relapsed B-cell lymphomas. [^18^F]FDG PET/CT plays a key role during staging and response assessment in patients with lymphoma; however, the evidence about its utility in CAR-T therapies for lymphomas is limited. This review article aims to provide an overview of the role of PET/CT during CAR-T cell therapy in B-cell lymphomas, focusing on the prognostic value of metabolic parameters, as well as on response assessment. Data from the literature report on the use of [^18^F]FDG PET/CT at the baseline with two scans performed before treatment started focused on the time of decision (TD) PET/CT and time of transfusion (TT) PET/CT. Metabolic tumor burden is the most studied parameter associated with disease progression and overall survival, making us able to predict the occurrence of adverse effects. Instead, for post-therapy evaluation, 1 month (M1) PET/CT seems the preferable time slot for response assessment and in this setting, the Deauville 5-point scale (DS), volumetric analyses, SUVmax, and its variation between different time points (∆SUVmax) have been evaluated, confirming the usefulness of M1 PET/CT, especially in the case of pseudoprogression. Additionally, an emerging role of PET/CT brain scans is reported for the evaluation of neurotoxicity related to CAR-T therapies. Overall, PET/CT results to be an accurate method in all phases of CAR-T treatment, with particular interest in assessing treatment response. Moreover, PET parameters have been reported to be reliable predictors of outcome and severe toxicity.

## 1. Introduction

Chimeric antigen receptor–engineered (CAR) T cells represent a new powerful tool for the treatment of refractory hematologic malignancies. The therapy is based on the genetic modification of patient-derived T cells by means of viral vectors enabling artificial chimeric antigen receptors expression to recognize specific tumor-associated antigens. The antigen CD19 is widely expressed on B-cell malignancies including non-Hodgkin lymphomas (NHL) such as diffuse large B-cell lymphoma (DLBCL), mantle cell lymphoma (MCL), and follicular lymphoma (FL), thus prompting the use of CAR-T cells targeting CD19 for the treatment of NHL [1].

Initially, CAR-T cells were developed for the treatment of acute lymphoblastic leukemia (ALL) and chronic lymphocytic leukemia (CLL) [2,3,4]; then, in 2017 two novel CD19-directed CAR-T cell products became commercially available for patients with relapsed/refractory (R/R) aggressive B cell NHL, comprising DLBCL, high-grade B cell lymphoma, primary mediastinal B cell lymphoma (PMBCL), and transformed FL and MCL, after two or more lines of therapy [5]. To date, four CAR T-cell therapies targeting the CD19 antigen on B cells have been approved by the Food and Drug Administration (FDA) and the European Medicines Agency (EMA): Axicabtagene Ciloleucel (Yescarta, Kite Pharma) [6,7]; Tisagenlecleucel (Kymriah, Novartis Pharmaceuticals) [8,9]; Brexucabtagene Autoleucel (Tecartus, Kite Pharma) [10,11]; and Lisocabtagene Maraleucel (Breyanzi, Juno Therapeutics) [12,13] (Table 1).

In later years, in fact, several multicenter studies on CAR-T for R/R NHL [14,15,16,17] have shown unexpected success in patients with R/R CD19+ B-cell malignancies. In particular, great results were shown concerning the overall response rate (ORR) and complete response (CR), with a major outcome benefit and durable response, compared to standard therapies.

Currently, CARs have been categorized into four “generations” according to the number of intracellular signaling molecules [18,19]. The first generation of CAR-T consists of the single intracellular signaling domain of CD3ζ, without the presence of a costimulatory signal. Instead, to increase the antitumor activity [20], second and third generations of CAR-T have incorporated, respectively, one or two co-stimulatory signaling domains in combination with the CD3ζ chain, most commonly CD28 or CD137 (4-1BB), and more [21,22,23]. Finally, fourth-generation “armored” CAR-T cells were manipulated to additionally express co-stimulatory ligands or cytokines, aiming to improve the tumoricidal effect of CAR-T cells [24].

CAR-T cells therapy comprises in general several phases: initially it starts with the apheresis of the peripheral blood mononuclear cells (PBMCs) of the patients, followed by the separation of T cells, for ex vivo transduction and expansion. The isolated T cells are then activated and transduced (e.g., using viral vectors) to express receptors for specific tumor antigens. At the end of the process it is necessary to expand the T cells that have been genetically engineered for obtaining a sufficient number of them. Normally, 4–6 weeks is spent between leukapheresis and the CAR-T administration in which patients often receive a “bridging therapy” to control their disease. Furthermore, patients usually receive conditioning chemotherapy just before the CAR-T reinfusion, called “lymphodepletion chemotherapy”, usually using cyclophosphamide (Cy) alone or Cy combined with fludarabine (FC) with the aim to reduce the lymphoma burden and increase the efficacy of CAR-T therapy. Finally, the last phase consists of thawing and infusion [25].

As for other lines of treatment in lymphomas, imaging attains an essential role for the evaluation of the patients before and during CAR-T cell therapy. In this context, [^18^F]Fluorodeoxyglucose Positron Emission Tomography/Computed Tomography, i.e., [^18^F]FDG PET/CT, has maintained its importance, although the number of publications specifically dedicated to lymphoma imaging for CAR-T is limited [26]. In the present article, we aim to illustrate the literature data and the major findings related to [^18^F]FDG PET/CT use during CAR-T cell therapy in B-cell lymphomas.

## 2. [^18^F]FDG PET/CT in Lymphomas Treated with CAR-T Cells

[^18^F]FDG PET/CT imaging contributes significantly both to disease staging, in association with clinical, morphological, and histological data, and for the response assessment of lymphoma patients, thus the imaging is becoming an important part of successful treatment management strategies [27]. With the rapid development of CAR-T cells, monitoring response therapy has become essential to identify treatment failure as early as possible and to correctly differentiate between responders and non-responders, with a longer and shorter predicted OS, respectively.

**Figure 1:**
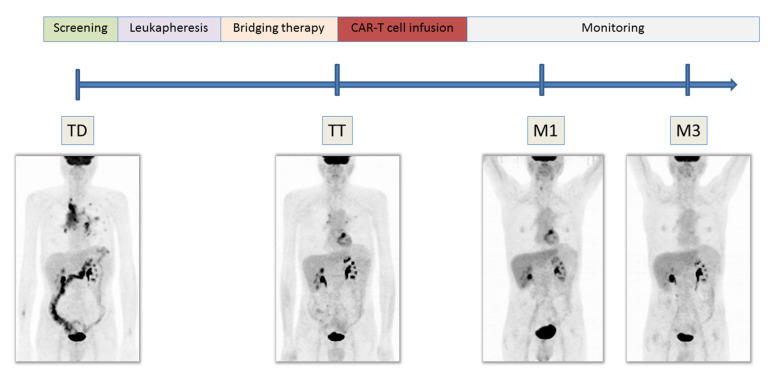
Schematic illustration of the time line for CAR-T cell therapy and corresponding schedules for [^18^F]FDG PET/CT imaging, respectively at TD (time of decision), TT (time of transfusion), M1 (1 month after CAR-T cell infusion), and M3 (3 months evaluation after CAR-T cells infusion).

In the context of CAR-T therapy, patients are usually investigated with a biopsy to evaluate CD19 status and [^18^F]FDG PET/CT at four relevant timings (Figure 1). Two scans are obtained before the infusion of the CAR-T cells: at the time of decision (TD) PET/CT, used to evaluate the next therapeutic approach, and the time of transfusion (TT) PET/CT, performed immediately before the CAR-T infusion. Subsequently, two PET/CT scans are used to monitor the response to therapy: 1 month (M1) PET/CT and 3 months (M3) PET/CT, showing a sensitivity and specificity of 99% and 100%, respectively, at the time of the first response assessment [28,29]. Several PET/CT scans do not represent a problem for patients. Furthermore, the EANM guidelines provide some recommendations to reduce FDG administration, such as the increase of study/bed duration and the use of modern PET/CT systems, with a higher sensitivity or an improved performance using new enhanced technology [30].

However, in the clinical setting, baseline PET/CT is typically obtained only at TD PET/CT, since not all centers perform both pre-therapy scans. Therefore, it remains uncertain whether an early response assessment performed after the CAR-T infusion represents a response to bridging or lymphodepleting therapy, or whether it represents the effective patient’s response to CAR-T therapy. Consequently, the optimal imaging timing of during CAR-T therapy is not fully determined yet. On the other hand, bridging therapy itself remains an area of discussion [31].

Notably, when it comes to the interpretation of the imaging data, several PET parameters have been used in clinical setting, i.e., the Deauville 5-point scale (DS), SUVmax, and its variation between different time points (∆SUVmax), as well as volumetric analyses. The DS is an international scale to visually compare the uptake of lymphoma lesions with referring tissues, such as the liver and the mediastinum. At the time of the response assessment, an uptake greater than that of the liver is usually used as the cut-off for the definition of an unfavorable response [32]. SUVmax can be measured as a single value in post-therapy PET/CT and/or at different time points with the ∆SUVmax method of the “hottest” tumor lesion. An unfavorable response herein, as confirmed in several studies, is defined as when the reduction in the SUVmax is inferior or equal to 66% [33,34,35,36,37]. Instead, the volumetric analysis implies the computation of the metabolically active lymphoma volume (MTV) with various methods [38,39], which can be used as a prognostic factor at the baseline before the treatment starts, or at the M1 evaluation (Table 2).

To differentiate the responders from non-responders, Cohen et al. [49] proposed the use of the DS and ΔSUVmax methods. A Deauville score > 3 on M1-PET/CT scans and the ΔSUVmax method at one month after the CAR-T infusion using TD-SUVmax as the reference for comparison (and not to TT-SUVmax), were found as the strongest predictors of a short OS. The use of the DS scale at M1-PET/CT was also validated by Jean Galtier et al. [54], demonstrating the strong impact of the quality of response according to the DS scale on the outcome of the patients, as recently confirmed by Kuhnl et al. [50] who demonstrated that patients with a DS3-4 response had a risk of an early relapse of 31%. Instead, Breen et al. [31] analyzed the value of SUVmax at M1 PET/CT as a predictive parameter, and considered the threshold of 10 as a significant prognostic predictor for patients with partial response/stable disease (PR/SD). It was subsequently confirmed by Al Zaki et al. [55], which supported an SUVmax value > 10 as a useful biomarker at M1 PET/CT post-CAR-T cells infusion.

The quantitative parameters from PET/CT in patients treated with CAR-T cells have been also evaluated by Shah et al. [40] in seven patients with DLBCL and FL. At M1 PET/CT, three of the patients showed no measurable MTV, two patients had an increase in MTV, and two had residual MTV. Only those patients without residual MTV remained in CR during the follow-up.

Despite the small cohort, they demonstrated that the M1 PET/CT assessment may distinguish responders from non-responders and specifically long-term responders.

In fact, long-term responders showed CR at M1 PET/CT, related to the rapid and robust onset of the response. On the contrary, M1 PET/CT clearly showed residual involved sites in PR or signs of early progression in non-responders.

In this setting, M1 evaluation seems the preferable time slot for response assessment and may be used to trigger a possible therapeutic intervention.

The importance of adequate disease control before the CAR-T has been recently shown by Bailly et al. [51] in R/R NHL patients. In their study, all patients underwent TD and TT PET/CT imaging and most of them received salvage chemotherapy before leukapheresis or bridging therapy before the CAR-T administration. On TT PET/CT, 50% of patients presented CR or PR according to the Lugano Criteria [27], and they showed longer event-free survival (EFS) in comparison to the others. Moreover, five of the seven patients who didn’t receive salvage therapy before the CAR-T administration experienced an early relapse. Their EFS curve confirmed that the metabolic response on TT PET/CT could successfully predict the response to CAR-T cells. These data were confirmed by Sesquez et al. [46], who demonstrated the use of MTV, expressed as ∆MTVpre-CAR-T, and TLG, expressed as ∆TLGpre-CAR-T, during bridging therapy before lymphodepletion, as a significant prognostic factors for predicting EFS, meaning that patients with satisfactory disease control before lymphodepletion had an overall longer PFS. Instead, in a cohort of 27 patients with R/R classical Hodgkin lymphoma (cHL) treated with lymphodepletion and CAR-T cells, Voorhees et al. [52] showed that a high MTV before lymphodepletion and the CAR-T cell infusion was associated with inferior PFS. On the contrary, receiving bridging therapy did not show any difference in PFS.

### 2.1. Metabolic Parameters at Baseline [^18^F]FDG PET/CT

Several groups have explored the predictive/prognostic value of metabolic parameters in patients treated with CAR-T therapy and, especially, the role of baseline [^18^F]FDG PET/CT scans in predicting the outcome and thus assisting in the selection of patients benefiting from the treatment. This is important because a consensus statement to guide the management and treatment of patients relapsing after CD19 CART cell therapy has not yet been achieved. Byrne et al. differentiated patients with an early relapse who maintain CD19+ that could beneficiate from a PD-1 inhibitor, from patients with a late relapse which can be considered for other salvage modalities, including reinfusion of the CAR-T cells, ibrutinib, immunomodulatory therapy, or, if the tumor is localized, radiation therapy [56].

Cohen et al. [49] demonstrated that a shorter OS was associated to the TD-SUVmax > 17.1, which resulted in an independent risk factor. Moreover, its combination with ECOG performance status and serum LDH levels could help clinicians to identify non-responders at staging evaluation.

In the literature, there has also been reported a particular interest in metabolic tumor burden measured on pre-treatment PET/CT that can be correlated with disease progression and the OS; Vercellino et al. [43] assessed that a high total MTV (TMTV) obtained on the TD and TT scans was one of the most discriminating outcome factors, especially for an early relapse prediction. They also associated high TMTV (>80 mL) with the number of extranodal (EN) sites > two to create a risk model, thus identifying three groups of patients having different outcomes. These findings were also consistent for Dean et al. [42] who demonstrated that a lower tumor burden (an MTV cut-off value of 147.5 mL) had a stronger correlation with prolonged OS and PFS in two cohorts of patients with a different follow-up and treatment (cohort one included patients enrolled on a prospective clinical trial, which prohibited the use of bridging therapy).

Hence, recurrence after CAR-T was observed in more than half of patients; in this setting, PET/CT imaging could predict lesions at a high risk of local failure, including high metabolic activity, size, or location (EN) [45].

However, not all studies in the literature found the baseline metabolic parameters to be significant prognostic factors. Bailly [51] and Wang [41] showed that, although the metabolic parameters were significantly higher in the non-responder patients, the prognostic value of the TT MTV/TLG on the response to treatment or the EFS of the tumor burden was not confirmed on the logistic regression model. This is partially due to the small cohort of patients’ population (40 and 19 patients, respectively). The prognostic impact during the CAR-T cell therapy was also evaluated by Iacoboni in a series of 35 patients [48]. Therein, the baseline TMTV with a cut-off of 25 cm^3^ was associated with a lower PFS and a shorter OS, but without a significant difference, maybe influenced by the short follow-up (median follow-up of 7.6 months).

### 2.2. Unconventional Responses: Pseudoprogression

The assessment of the response to CAR-T cell therapy with [^18^F]FDG PET/CT can be challenging, since it can be difficult to distinguish the tumor sites of inflammation from the disease progression, which consequently leads to false-positive findings.

In fact, the manifestation of pseudo-tumor progression during CAR-T cells therapy is completely overlapping to the previously reported patient’s response patterns during immunotherapy with checkpoint inhibitors [57].

Despite the possible association with CAR-T activity and long-term disease control, the causes of pseudoprogression remain unknown and many studies are necessary to describe the extent of this phenomenon.

With this regard, Danylesko et al. [58] have assessed the relationship between the serum interleukin-8 (IL-8) level and M1 PET/CT response to CAR-T in 56 adult patients with R/R NHL. The IL-8 level is a marker of tumor burden and allows us to monitor the response to vemurafenib and ipilimumab in metastatic melanoma and non-small lung carcinoma patients [59,60]. From the study, 14/56 patients showed “signs of suspected progression” with a deterioration of clinical status starting in 7–10 days following the CAR-T cells infusion; among them, only 11/14 cases showed a real progression. In those three pseudoprogressive diseases, the IL-8 levels were low or had decreased on day 14 in parallel with the beginning of the clinical response. Conversely, patients showing a true early progression had high serum IL-8 levels. At M1 [^18^F]FDG PET/CT, in pseudoprogressive patients there was a partial resolution of the previous FDG-avid lesions consistent with a PR or no-FDG avid residual mass compatible with the complete response (CR) [58].

A tumor flare and local immune activation were reported by Wang et al. [41] on the PET/CT scanning on three NHL patients 1 week after CAR-T cell therapy. They suspected that the increased metabolic activity was associated with immune cell infiltration, given an unexpected lymphoma dimensional growth in a short period, and subsequently a rapid regression. To confirm the visual assessment, SUVmax, MTV, and TLG (total lesion glycolysis) were measured at the baseline (MTV 149.6 cm^3^, SUVmax 23.5, and TLG 1421.1) 5 days after the infusion and during local complications (MTV 298.1 cm^3^, SUVmax 21.8, and TLG 5316) and at M1 (MTV 0.76 cm^3^), confirming the complete resolution of the lesion at M1 PET/CT. A very similar event of pseudoprogression was described by Boursier et al. [61] in a case report of a woman with DLBCL. Herein, 8 days after CAR-T cell therapy, the PET/CT examination showed an early flare-up phenomenon associated to clinical deterioration and new nodal involvement and progression, yet also associated with a decrease in MTV in the primitive lesion. The M1 PET/CT confirmed the lack of real progression, with regression of the previously described finding.

The phenomenon of “pseudoprogression” was confirmed by Cohen et al. [62] also in a 23-year-old female patient with R/R DLBCL. Nine days post-infusion, the patient complained of dyspnoea worsening and presented with enlarged cervical masses. Fifteen days post-infusion, on [^18^F]FDG PET/CT imaging, all the involved sites demonstrated an increase in both FDG-uptake (SUVmax 22.8 versus SUVmax baseline 14.5) and size, with additional new FDG-avid findings. To differentiate between progressive metabolic disease and pseudoprogression, a cervical lymph node biopsy was performed with the histological report of ‘’Inflammatory changes without evidence of lymphoma’’. Forty-two days post-infusion, the imaging findings disappeared or improved (∆SUVmax > 66%), indicating a complete metabolic response and supporting the hypothesis of pseudoprogression.

Of note, the pseudoprogression reported during the CAR-T therapy was more rapid, both in onset and duration, compared with the conventional checkpoint inhibitors. This phenomenon could be an indirect sign of a greater efficacy of CAR-T cell therapy.

At the same time, it is fundamental to predict life-threatening consequences produced by an enlargement and local compression in the staging evaluation. Therefore, PET/CT might be useful in the identification of potentially dangerous sites of involvement and estimate PET-parameters to take precautions in preventing damages in local organs [41].

## 3. [^18^F]FDG PET/CT and Toxicity during CAR-T Cell Therapy

Adverse effects are associated with all cancer therapies, and CAR-T cells are no exception. In cytotoxic chemotherapy, adverse effects are off-target with the permanent genetic modifications of cells in the entire organism and long-term clinically significant consequences; in CAR-T cells infusion, the major toxic manifestations are on-target effects and depend on the specificity of antibody single-chain variable fragments and T-cell activation. These toxic effects are thus reversible when the target cell is removed, or the engraftment of the CAR-T cells is broken off [63,64].

Primarily, cytokine release syndrome (CRS), neurologic toxicity, and coagulation disorders are all serious limitations in the improvement of CAR-T, particularly in adult ALL patients with more severe manifestations in comparison with patients with other B cell malignancies [65].

The onset of adverse effects is a challenge in CAR-T treatment and the evaluation of baseline PET/CT could have a prominent role through the use of different parameters. Derlin et al. [47] found that higher lymphoma metabolic activity, measured with SUVmax, was associated with neurotoxicity. Of note, in DLBCL patients, the value of SUVmax reflects indirectly the Ki-67 proliferation index, and therefore tumor aggressiveness [66]. Thus, patients with high Ki-67, and consequently high SUVmax and potential aggressive lymphoma, may be particularly inclined to the manifestation of toxicity.

The following paragraphs will undergo more in details on the specific adverse events during CAR-T cell therapy and the role of [^18^F]FDG PET/CT imaging in their occurrence.

### 3.1. Cytokine Release Syndrome (CRS)

CRS is the most frequent complication of CAR-T cell therapy, occurring within 1–10 days post-infusion and usually lasting for up to 9 days. The crucial role of its pathogenesis is the activation of activated myeloid cells [67].

CRS can vary from mild to life threatening, although its occurrence is not seen in all patients. The syndrome usually starts with a fever, followed by hypotension, respiratory distress, capillary leak syndrome, and neurological disorders including a seizure and/or multiorgan toxicity. The first line therapy consists in the administration of steroids and tocilizumab [68].

According to our knowledge, there are no cases of patients who have performed PET/CT during CRS. The choice to carry out the follow-up PET after 1 month of infusion has influenced this aspect, since it did not confound findings with the development of CRS, without false-positive results due to inflammation [40]. However, the data available in the literature report for the [^18^F]FDG PET/CT parameters, already at the baseline, have the capability to predict the occurrence of CRS. In the multicenter studies investigating CAR-T for R/R NHL (ZUMA-1, JULIET, and TRANSCEND) [14,15,16], the tumor burden was strictly correlated with CRS’s severity, as recently described by Hong et al. [44], who assessed at staging PET/CT that a high tumor burden, reflected by SUV average (SUVavg), MTV, and TLG, was a significant risk factor to develop any grade of CRS. This finding was confirmed by Dean [42] and Wang [41] and, in particular, the latter found different cut-off values of MTV and TLG, according to CRS grading. Although, this association has not been found with neurotoxicity. In disagreement with these previous studies, Derlin [47] and Zhou [53] observed no association between [^18^F]FDG PET/CT parameters and CRS, mainly due to the relatively limited cohort of patients and the number of high-grade toxicities.

### 3.2. Immune Effector Cell Associated Neurotoxicity Syndrome (ICANS)

The neurotoxicity associated with CAR-T cell therapy remains idiosyncratic and poorly characterized. This consists of a variable spectrum of neurophysiological dysfunction arising in CAR-T cells after 1 week of infusion. The most frequent clinical manifestations are encephalopathy, a confusional state, delirium, or changes in mental-status up to coma [5,69]. This syndrome is defined as immune effector cell-associated neurotoxicity syndrome (ICANS) and evaluated with a consensus tool for encephalopathy grading (ICE score) by the American Society for Transplantation and Cellular Therapy (ASTCT) [70].

In a patient with suspected ICANS, the first step consists of excluding mimics or other conditions such as infection, cerebral involvement of B cell malignancy, drug toxicity, metabolic derangement, or organ dysfunction. Morphological brain imaging such as computed tomography (CT) and magnetic resonance imaging (MRI) are mostly normal in mild ICANS and even in more severe ICANS [71].

Actually, brain [^18^F]FDG PET/CT may play a prominent role also in the detection of this syndrome, exploiting the evaluation of metabolic activity in the brain, as already carried out in other conditions of metabolic distress and neuronal injuries [72,73,74].

Even in sites where MR imaging showed no alteration, focal deficits have been detected with [^18^F]FDG PET/CT as areas of hypometabolism, or as patterns of elevated velocities on a transcranial Doppler ultrasound. This finding was demonstrated by Rubin et al. [73], who characterized the neurological CAR-T cells toxicity which was associated up to 2 months post-transfusion. Of their cohort, six patients presenting with neurotoxicity associated with abnormal EEGs were examined with brain PET/CT scans. In five patients, the areas of the EEG alterations corresponded to hypometabolic areas on [^18^F]FDG PET/CT. Moreover, in three of these patients, a transcranial Doppler ultrasound proved an elevated flow velocities correlated with the regions of PET hypometabolism. These findings did not correlate with structural lesions and remained without elucidation of possible etiology.

Other few studies have mentioned the absent correlation between functional and morphological imaging. Recently, Paccagnella et al. [75] described a DLBCL male with grade-2 ICANS that developed at 4 days after the CAR-T infusion. [^18^F]FDG PET/CT of the brain showed a global hypometabolism, especially involving the left hemisphere and the frontal regions with the clinical manifestation of the ideo-motor slowing, nominal aphasia, slowing fluency, frontal release signs, postural myoclonic tremor, and paligraphia. On the other hand, despite the diffuse slowing as depicted on the EEG, the MRI showed the absence of pathological involvement. After intravenous steroid administration, the patient responded completely.

These findings are consistent with the recently published case report from Vernier et al. [76] in which after 2 days of infusion, a DLCBL patient showed CRS followed by neurotoxicity syndrome (slowness, comprehension disorders for complex orders, major ideational and ideomotor apraxia, static and kinetic cerebellar syndrome, and proprioceptive ataxia). Once again, diffuse EEG abnormalities were associated with no alterations on the brain MRI. At the 14th day of infusion, a [^18^F]FDG PET/CT brain scan was performed showing a bilateral and diffuse hypometabolism, predominantly in the parietal and temporal cortex, excluding the limbic and cerebellar lobes. Four months after CAR-T cell infusion, a new brain PET/CT showed only prefrontal and temporal hypometabolism. As Vernier [76] suggested, the presence of hypometabolism on PET with the concomitant lack of abnormal MRI findings is the key in CAR-T cell neurotoxicity evaluation.

Therefore, during the acute phase, PET/CT monitoring allowed for an early detection of cortical impairments correlating with neurological alterations. In the remission phase, the PET findings showed a parallel improvement of the clinical symptoms, suggesting the reversibility of CAR-T cell-related neurotoxicity.

Metabolic imaging therefore represent an important tool in lymphoma setting. However, during PET/CT interpretation, it is important to remember all conditions in which FDG uptake is induced by others processes, such us physiologic uptake (brain, kidney, and urinary tract), infectious and inflammatory disease, benign cancer, and artifacts. Moreover, all situations that can lead to false-negative FDG interpretation, such as small size lesions, hyperglycemia/hyperinsulinemia, and recent therapy [77].

## 4. Conclusions

The unique features of [^18^F]FDG PET/CT make it the most valuable imaging tool in CAR-T patients. It turned out to be an accurate method for assessing the extent of the lymphoproliferative process, resulting also in the selection of this treatment. Moreover, at the baseline, the PET parameters are predictive of the outcome and may identify a subgroup of patients mostly benefiting from CAR-T infusion from those tending to have a poorer prognosis and thus less therapeutic benefit. Due to its functional character, M1 PET/CT, and consequently PET parameters, play a central role in response assessment and differential diagnosis between progression and “pseudoprogression”. Last but not least, [^18^F]FDG PET/CT appears to show promise in predicting the risk of severe toxicity with recent growing interest of PET/CT brain scans in the evaluation of ICANS.

## Figures and Tables

**Table 1:** List of approved Anti-CD19 CAR-T by the FDA and EMA.

Agency	Drug	Product/Campany	Indications
FDA	Tisagenlecleucel	Kymriah (Novartis Pharmaceuticals Corp.)	Adult R/R LBCL; Pediatric and young adult R/R B-cell precursor ALL
	Axicabtagene ciloleucel	Yescarta (Kite Pharma, Inc.)	Adult R/R FL; Adult R/R LBCL;
	Brexucabtagene autoleucel	Tecartus (Kite Pharma, Inc.)	Adult R/R B-cell precursor ALL; Adult R/R MCL
	Lisocabtagene maraleucel	Breyanzi (Therapeutics, Inc.)	Adult R/R LBCL
EMA	Tisagenlecleucel	Kymriah (Novartis Pharmaceuticals Corp.)	Pediatric and young adult R/R B-cell ALL; Adult R/R DLBCL and R/R FL.
	Axicabtagene ciloleucel	Yescarta (Kite Pharma, Inc.)	Adult R/R DLBCL; Adult R/R PMBCL; Adult R/R FL
	Brexucabtagene autoleucel	Tecartus (Kite Pharma, Inc.)	Adult R/R MCL
	Lisocabtagene maraleucel	Breyanzi (Therapeutics, Inc.)	Adult R/R DLBCL; Adult R/R PMBCL; Adult R/R FL3B

Abbreviations: R/R = relapsed or refractory; ALL = B-cell precursor acute lymphoblastic leukemia; FL = follicular lymphoma; MCL = mantle cell lymphoma; DLBCL = diffuse large B-cell lymphoma; LBCL = large B-cell lymphoma; PMBCL = primary mediastinal large B-cell lymphoma; FL3B = follicular lymphoma grade 3B. *Source:* https://www.fda.gov/drugs/resources-information-approved-drugs/oncology-cancer-hematologic-malignancies-approval-notifications and https://www.ema.europa.eu/en/medicines (accessed on 29 March 2022).

**Table 2:** Summary of available studies investigating [^18^F]FDG PET/CT in lymphoma treated with CAR-T.

Authors	Study Type	Patients Number	Histologic Subtype	Treatment	Response Criteria	Main Findings
Shah et al. [40] 2018	Prospective	7	DLBCL, FL	CTL019 CAR-T	Lugano	Patients with no residual MTV at M1 PET/CT remained in remission > 2 years post-treatment
Wang et al. [41] 2019	Retrospective	19	DLBCL, FL	CD19 CAR-T	PERCIST	Higher disease burden at baseline (MTV and TLG) was associted with a higher risk of severe CRS (grade 3 to 4)
Dean et al. [42] 2020	Retrospective	96	LBCL, PMBCL	CD19 CAR-T	Clinical response	Baseline MTV is associated with OS and PFS
Vercellino et al. [43] 2020	Retrospective	116	DLBCL	CD19 CAR T	Lugano	TMTV resulted as one of the risk factors for early progression
Hong et al. [44] 2021	Retrospective	41	NHL	CD19 CAR-T	Lugano	Early post-therapy SUVavg and MTV resulted independent risk factors to OS and PFS. High baseline tumor burdens resulted significantly associated to increased CRS and cytokine levels.
Figura et al. [45] 2021	Retrospective	63	DLBCL, FL, PMBCL	CD19 CAR-T	Cheson	Lesions at increased risk of local failure resulted with diameter ≥ 5 cm, SUVmax ≥ 10, or extranodal
Sesques et al. [46] 2021	Retrospective	72	DLBCL, PMBCL, trFL, trMZL	CD19 CAR-T	Lugano	Metabolic volume kinetics before CAR T resulted superior to initial tumor bulk for the prediction of the PFS, whereas SUVmax (cut-off 14) at first post-CAR-T evaluation resulted independently related to OS.
Derlin et al. [47] 2021	Retrospective	10	DLBCL, trFL,	CD19 CAR-T	Lugano	To obtain remission an early metabolic response at M1 is required. Poor outcome was associated with an early suppression of the metabolic activity in lymphoid organs, such as spleen or lymph nodes
Iacoboni et al. [48] 2021	Retrospective	35	LBCL	CD19 CAR-T	Lugano	High baseline TMTV (≥25 cm^3^) was associated with shorter PFS
Ruff et al. [28] 2021	Retrospective	43	LBCL	CD19 CAR-T	Lugano	At the time of first response assessment, lesional sensitivity and specificity were 99% and 100%, respectively
Cohen et al. [49] 2022	Retrospective	48	DLBCL	CD19 CAR-T	N/A	At M1 post-CAR-T DS > 3 and ΔSUVmax ≤ 66% were predictive to OS.
Kuhnl et al. [50] 2022	Retrospective	171	LBCL	CD19 CAR-T	DS	DS response at M1 PET/CT was independent predictor to time to relapse
Bailly et al. [51] 2022	Retrospective	40	NHL	CD19 CAR-T	Lugano,	FDG-PET pre-infusion predicted EFS and CAR-T cells response.
Voorhees et al. [52] 2022	Prospective	27	HL	CD30 CAR-T	Lugano	Shorter PFS was associated with a high MTV before lymphodepletion and CD30 CAR-T cell infusion.
Zhou et al. [53] 2022	Retrospective	24	DLBCL, BL, trFL	CD19/CD22 CAR-T	N/A	Radiomic features at baseline could predict the PFS and OS.

Abbreviations: DLBCL = diffuse large B-cell lymphoma; FL = follicular lymphoma; HL = Hodgkin lymphoma; DS = Deauville score; SUV = standardized uptake value; MTV = metabolic tumor volume; TMTV = total metabolic tumor volume; TLG = total lesions glycolysis; ΔMTV/ΔTLG/ΔSUV = variation of MTV/TLG/SUV; R/R NHL = relapsed/refractory non-Hodgkin lymphoma; PMBCL = primary mediastinal B-cell lymphoma; trFL = transformed follicular lymphoma; trMZL = transformed marginal zone lymphoma; LBCL = large B-cell lymphoma; PFS = progression-free survival; OS = overall survival; M1 PET/CT = 1 month PET/CT; EFS = event-free survival; and N/A = not applicable.
